# Supplementary material for: Empirical methods for controlling false positives and estimating confidence in ChIP-Seq peaks
Source: BMC Bioinformatics. 2008 Dec 5;9:523. doi: 10.1186/1471-2105-9-523 (PMC2628906; doi:10.1186/1471-2105-9-523)
Supplement: Additional File 1 — A variety of html documents from the USeq web site detailing the available applications, their best usage, output file type descriptions, command line menus, etc. [file 1471-2105-9-523-S1.zip › USeqUserGuides/JavaDoc.zip!/JavaDoc/index-files/index-15.html]

 
 
 
 
 
 
P-Index
 


 

 
function windowTitle()
{
    parent.document.title="P-Index";
}
 
 
 

 

 


 
   
  
 
 
 
   
 
   
          Overview     
        Package   
        Class   
        Use   
          Tree     
          Deprecated     
       Index    
          Help     
   
 
 
  
 
 
 

 
  
   PREV LETTER   
   NEXT LETTER    
  
    FRAMES     
   NO FRAMES     
  
    All Classes  ');
  }
  //-->
 
 
    All Classes  
 


  
 
 
  
 

 A   B   C   D   E   F   G   H   I   J   L   M   N   O   P   Q   R   S   T   U   V   W   X   Z   
    
 P  
 
   paintComponent(Graphics)   - 
Method in class gata.geneGlyphs. GlyphPanel 
 Watch order of drawing, optimize to avoid changing stroke
   paintComponent(Graphics)   - 
Method in class gata.plotter. AlignPanel 
  
   paintComponent(Graphics)   - 
Method in class trans.graphics. IntervalDrawPanel 
  
   paintComponent(Graphics)   - 
Method in class trans.graphics. RankedSetDrawPanel 
  
   paintComponent(Graphics)   - 
Method in class trans.graphics. VirtualCelPanel 
  
   paintComponent(Graphics)   - 
Method in class util.apps. ScatterDrawPanel 
  
   paintComponent(Graphics)   - 
Method in class util.bio.cluster. ClusterDrawPanel 
  
   pairedLogRatios(float[][], float[][])   - 
Static method in class util.gen. Num 
 Given two float[replica][values] for treat and cont, for each replica makes matched pairwise log ratios 
 between treat and control.
   pairedRatios(float[][], float[][])   - 
Static method in class util.gen. Num 
 Given two float[replica][values] for treat and cont, for each replica makes matched pairwise ratios 
 between treat and control.
   pairedRelativeDifferences(float[][], float[][])   - 
Static method in class util.gen. Num 
 Given two float[replica][values] for treat and cont, for each replica makes matched pairwise relative differences 
 between treat and control.
   pairwiseCorrelationScore(ExpressedGene[])   - 
Method in class expr. CorrelationMaps 
 Average coorelation coefficient * square root of number of genes.
   pairwiseMeans(float[])   - 
Static method in class util.gen. Num 
 Returns all pairwise averages.
   pairwiseMeansDouble(float[])   - 
Static method in class util.gen. Num 
 Returns all pairwise averages.
   paramsPresent()   - 
Method in class bioroot. LoginBean 
  
   paramsPresent()   - 
Method in class cgar.beans. LoginBean 
 Look for params.
   ParseAgilentData   - Class in  expr  Parses Agilent data based on a list of column names.   ParseAgilentData(String[])   - 
Constructor for class expr. ParseAgilentData 
  
   parseAgilentFile(File)   - 
Method in class expr. ParseAgilentData 
  
   parseAgilentFile()   - 
Method in class trans.misc. ConvertAgilentData 
  
   parseAndLoad(File)   - 
Method in class edu.utah.seq.parsers. ElandMultiParser 
  
   parseAndPrint(File)   - 
Method in class util.bio.parsers. Cap3Parser 
  
   parseAndPrintContig(ArrayList)   - 
Method in class util.bio.parsers. Cap3Parser 
  
   parseAndPrintMatches()   - 
Method in class expr. FileCrossFilter 
  
   parseAndPrintMatches()   - 
Method in class expr. FileMatchJoiner 
  
   parseBl2SeqResults(String[])   - 
Method in class util.bio.wrappers. BL2Seq 
 This takes an array of BLAST bl2seq results and parses the info into an array
   parseBlast(String[])   - 
Method in class gata.aligner. Blast 
  
   parseBlast(String[])   - 
Static method in class util.bio.wrappers. SimpleBlast 
  
   parseBlastGeneral(String[])   - 
Static method in class util.bio.wrappers. SimpleBlast 
 Parses blast output from blastall for dna into BlastResult objects.
   parseChromData(String, int[][])   - 
Method in class edu.utah.seq.parsers. ElandSequenceParser 
  
   parseChromFile(File, int[][])   - 
Method in class edu.utah.seq.parsers. ElandSequenceParser 
  
   parseChromosomeName(String)   - 
Static method in class trans.misc. Util 
 Attempts to parse a chromosome name.
   parseColumn(File, int)   - 
Static method in class util.gen. IO 
 Parses the indexed column of a tab delimited file, all lines included.
   parseControlGeneFile(File)   - 
Static method in class trans.roc. ControlGene 
 parses a control gene file
 ex: #name      chrom   strand  exonStarts      exonEnds        notes
 ex: NM_001035267       chr12   +       54796640,54797239,54797532,     54796850,54797262,54797882,     Z12962_Homologue to yeast ribosomal protein L41_H
   parseCyberT(File)   - 
Method in class trans.main. ReplaceWindowScores 
 Parses a file for a tab delimited list of at minimum, chrom, start, stop, (optional) notes into a
 GenomicRegion[].
   parsed()   - 
Method in class edu.utah.seq.parsers. ElandExportLine 
  
   parseDataLines(File)   - 
Method in class edu.utah.seq.methylation454. MethylationParser 
  
   ParseDecodeGenotypeData   - Class in  util.bio.parsers  Parses a directory of joined decode data files, ie 'chr10       358624  3       0       0.992874        cnv1071p9' chr,pos,#A,#B,1-pval,SNPName.   ParseDecodeGenotypeData()   - 
Constructor for class util.bio.parsers. ParseDecodeGenotypeData 
  
   parseDNAFileName(String)   - 
Static method in class gata.main. GATAUtil 
  
   parseDoubles(String[])   - 
Static method in class util.gen. Num 
 Parses doubles from a String[] of doubles.
   parseFasta()   - 
Method in class util.bio.parsers. FetchSeqsQualsByName 
  
   parseFields()   - 
Method in class util.apps. HouseBean 
  
   parseFile(File)   - 
Method in class util.apps. Flash 
 Strips out " that stupid excel adds in.
   parseFile(File, int, int)   - 
Static method in class util.bio.annotation. Coordinate 
 Parses a tab delimited chr,start,stop file.
   parseFile(File)   - 
Static method in class util.bio.wrappers. Primer3Pick 
 Parses a tab delimited file where the sequence to pick primers from is in column 0.
   parseFile(File, int, boolean)   - 
Static method in class util.gen. IO 
 Parses a tab delimited file, the indexed column is used as the key, 
 the entire line as the value, blank lines skipped, returns null if 
 a duplicate key is found.
   parseFile(String)   - 
Method in class util.xml. ParseXMLFile 
 Parses XML file and returns XML document.
   parseFixedStepWigFile(File, float)   - 
Static method in class trans.misc. Wig2Bar 
 Parses a fixedStep wig file, skips any track type data.
   parseGeneFile(File)   - 
Static method in class expr. CorrelationMaps 
 Converts a tab delimited file of (name chr start stop val1 val2 ...) into an ExpressedGene[]
   parseGeneTableFile(File, int)   - 
Method in class util.bio.parsers. UCSCGeneModelTableReader 
  
   parseGenomicRegionsFile(File)   - 
Static method in class trans.graphics. RankedSetAnalysis 
 Parses a regions file.
   parseGff(File)   - 
Method in class util.bio.parsers.gff. ParseWormBaseGffToUCSC 
  
   parseGrGraph(GrGraph)   - 
Static method in class trans.roc. Gr 
  
   parseInt(String, int)   - 
Static method in class util.gen. Num 
 Attemps to parse an int, returns failNumber if it fails.
   parseIntervalFile(File, int)   - 
Static method in class trans.anno. AnnotateRegions 
 Attempts to fetch a serialized array of Interval[], then sorts/ ranks the intervals by the median
 ratio of the sub window.
   parseIntervalFile(File, int)   - 
Static method in class trans.anno. AnnotateRegionsWithGeneList 
 Attempts to fetch a serialized array of Interval[], then sorts/ ranks the intervals by the median
 ratio of the sub window.
   parseIntervalFile(File)   - 
Static method in class trans.anno. IntersectRegions 
  
   parseInts(String[])   - 
Static method in class util.gen. Num 
 Parses ints from a String[] of ints.
   parseIt(File)   - 
Method in class edu.utah.seq.methylation454. MethylationParser 
  
   parseIt(File)   - 
Method in class edu.utah.seq.methylation454. ReadAlignParser 
  
   parseIt(File)   - 
Method in class trans.misc. ConvertGeoData 
  
   parseIt(File)   - 
Method in class util.bio.parsers.gff. Gff3Parser 
  
   parseIt(File)   - 
Method in class util.bio.parsers. MultiFastaParser 
 Every call adds to the list of sequences and names.
   parseIt(File)   - 
Method in class util.bio.parsers. RefSeqAnnotationParser 
  
   parseJobId(String[])   - 
Static method in class util.gen. Job 
 Attempts to parse 'Job Id:', returns null if not found.
   parseKeyValueGroups(String)   - 
Static method in class util.gen. Misc 
 Given a String of 'treat1=/file1,/file2*treat2=/file3,/file4*treat3=/file5,file6'
 Will return an ArrayList contining first a String[] of the keys {treat1,treat2,treat3}
 and second a String[][] of the parsed values matching the keys.
   parseKeyValues(String)   - 
Static method in class util.gen. Misc 
 Given a String of 'treat1=/file1,treat2=/file3, etc', no spaces,
 returns a LinkedHashMap of keys=values.
   parseLine(String, Pattern)   - 
Static method in class gata.aligner. Blast 
  
   parseLine(String, Pattern)   - 
Static method in class util.bio.wrappers. BL2Seq 
  
   parseLine(String, Pattern)   - 
Static method in class util.bio.wrappers. SimpleBlast 
  
   parseListingsFile()   - 
Method in class util.apps. ParseMLSHTML 
 Parses listing lines on 'List Price'
   ParseManipulateColumnDataForGeneSifter   - Class in  expr      ParseManipulateColumnDataForGeneSifter()   - 
Constructor for class expr. ParseManipulateColumnDataForGeneSifter 
  
   parseMemeText(String[])   - 
Method in class meme. MemeParser 
  
   parseMenu()   - 
Method in class util.apps.gump. Gump 
  
   ParseMiRNATable   - Class in  expr      ParseMiRNATable(String[])   - 
Constructor for class expr. ParseMiRNATable 
  
   parseMiRNATable()   - 
Method in class expr. ParseMiRNATable 
  
   ParseMLSHTML   - Class in  util.apps      ParseMLSHTML(String[])   - 
Constructor for class util.apps. ParseMLSHTML 
  
   parseMultiPartRequest(HttpServletRequest)   - 
Static method in class util.gen. HTML 
 Parses a multipart request returning a HashMap of fieldName: String, 
 if the same fieldName is found more than once, a String[] is created.
   parseNDF()   - 
Method in class trans.misc. ConvertNimblegenNDF2TPMap 
  
   parsePAIR()   - 
Method in class trans.misc. ConvertNimblegenPAIR2Cela 
  
   ParsePatternedWindows   - Class in  trans.roc  Finds RocWindows upstream and withing the neighborhood of the ATG start site of a patterned gene list.   ParsePatternedWindows(String[])   - 
Constructor for class trans.roc. ParsePatternedWindows 
  
   parsePicksFile(File, int)   - 
Static method in class trans.anno. AnnotateRegions 
  
   parsePicksFile(File, int)   - 
Static method in class trans.anno. AnnotateRegionsWithGeneList 
  
   parsePrimer3Results(String[])   - 
Method in class util.bio.wrappers. Primer3Wrapper 
 Takes the output of the primer3 app and breaks by record loading appriopriate Primer3Pick.
   parseQuality()   - 
Method in class util.bio.parsers. FetchSeqsQualsByName 
  
   Parser1lq   - Class in  trans.qc  Extracts control an pm lines from a text 1lq file.   Parser1lq(String[])   - 
Constructor for class trans.qc. Parser1lq 
  
   parseRegionFile(File)   - 
Static method in class trans.roc. ParseSgrsForParticularRegions 
  
   parseRegionFiles()   - 
Method in class trans.anno. IntersectKeyWithRegions 
  
   parseRegions(File)   - 
Static method in class trans.anno. Region 
 Parses a file for a tab delimited list of at minimum, chrom, start, stop, (optional) notes into a
 GenomicRegion[].
   parseRegionsFile(File)   - 
Static method in class trans.anno. IntersectRegions 
  
   parseResults()   - 
Method in class util.bio.wrappers. Segment 
  
   ParserForGff   - Class in  util.apps      ParserForGff()   - 
Constructor for class util.apps. ParserForGff 
  
   ParseSeqsFromUCSCGeneTable   - Class in  util.bio.seq      ParseSeqsFromUCSCGeneTable()   - 
Constructor for class util.bio.seq. ParseSeqsFromUCSCGeneTable 
  
   parseSgrFile(File, boolean)   - 
Static method in class trans.roc. MethodTester 
 Reads in an Sgr file.
   ParseSgrsForParticularRegions   - Class in  trans.roc  Returns oligo values that overlap particular regions.   ParseSgrsForParticularRegions(String[])   - 
Constructor for class trans.roc. ParseSgrsForParticularRegions 
  
   ParseSgrsForParticularScores   - Class in  trans.roc  Returns oligo values that overlap particular regions.   ParseSgrsForParticularScores(String[])   - 
Constructor for class trans.roc. ParseSgrsForParticularScores 
  
   parseStartStops(File, int, int)   - 
Static method in class util.bio.annotation. StartStop 
 Parses a tab delimited file (chr, start, stop, ...).
   parseStringArray(Object)   - 
Static method in class util.gen. HTML 
 For extracting either a String[] or making a new String[] by casting the object as a String.
   parseStringToDouble(String)   - 
Static method in class util.bio.parsers.gff. GffFeature 
  
   parseVariableStepWigFile(File, float)   - 
Static method in class trans.misc. Wig2Bar 
 Parses a variableStep wig file, skips any track type data.
   parseView(HttpServletRequest)   - 
Static method in class bioroot. Util 
 Parses a 12-49 type parameter named view to just the ints
   parseWigFileType(File)   - 
Static method in class trans.misc. Wig2Bar 
 Looks for the first line that begins with 'fixedStep' or 'variableStep', if none found returns null, otherwise returns one of those
 two names.
   parseWindowFile(File)   - 
Method in class igb.util. Windows2HeatMapSgr 
  
   ParseWormBaseGffToUCSC   - Class in  util.bio.parsers.gff      ParseWormBaseGffToUCSC(String[])   - 
Constructor for class util.bio.parsers.gff. ParseWormBaseGffToUCSC 
  
   ParseWormMart   - Class in  expr      ParseWormMart()   - 
Constructor for class expr. ParseWormMart 
  
   ParseXMLFile   - Class in  util.xml  This class represents short example how to parse XML file,
 get XML nodes values and its values.  
 It implements method to save XML document to XML file too   ParseXMLFile()   - 
Constructor for class util.xml. ParseXMLFile 
 Creates a new instance of ParseXMLFile
   partialPairwiseMeans(float[], float[])   - 
Static method in class util.gen. Num 
 Returns all pairwise averages between a and b but not self pairwise.
   Passwords   - Class in  util.gen  Methods for generating one time passwords.   Passwords()   - 
Constructor for class util.gen. Passwords 
  
   PatternMatch   - Class in  bioroot  Class to hold information and helper methods relating to pattern matches.   PatternMatch(int, int, String)   - 
Constructor for class bioroot. PatternMatch 
  
   PeakPicker   - Class in  edu.utah.seq.analysis  Use to find the peaks within EnrichedRegions   PeakPicker(EnrichedRegion[], File[], File[], int)   - 
Constructor for class edu.utah.seq.analysis. PeakPicker 
 Picks the best peak based on rescanning each enriched region using the defined window size.
   PeakPicker   - Class in  trans.main  Finds peaks in   Interval  s, both flat topped or peaks with sloping sides.   PeakPicker()   - 
Constructor for class trans.main. PeakPicker 
  
   PearsonCorrelation   - Class in  util.gen  Calculates Pearson Correlations (R) in two ways, using a straight static method on two arrays or
 by 1st adding paired arrays sequentially and 2nd calculating the correlation on the cumulative 
 sum.   PearsonCorrelation()   - 
Constructor for class util.gen. PearsonCorrelation 
  
   pentamerGenerator()   - 
Static method in class util.bio.xamer. XamerCounter 
  
   percent(int, int)   - 
Static method in class trans.main. OverlapCounter 
  
   percentile(Quantile[], double)   - 
Static method in class trans.cel. QuantileNormalization 
 Returns the value of a given percentile from a SORTED Quantile array.
   percentile(float[], double)   - 
Static method in class util.gen. Num 
 Returns the value of a given percentile from a SORTED array.
   permutate()   - 
Method in class trans.main. RandomizeLabels 
 Randomly permutates the ArrayName[].
   permutate()   - 
Method in class util.apps. IntersectLists 
  
   pick(int)   - 
Method in class edu.utah.seq.data. PointData 
 Randomly picks the numToPick from the position and score arrays and replaces.
   pickPeaks()   - 
Method in class edu.utah.seq.analysis. EnrichedRegionMaker 
 Attempts to pick the best peak within each enriched region.
   pickPeaks()   - 
Method in class edu.utah.seq.analysis. PeakPicker 
 Main method of class.
   pickPeaks(Interval[])   - 
Method in class trans.main. PeakPicker 
  
   pickRandomFragments(String, String, int)   - 
Method in class edu.utah.seq.data. Simulator 
 Picks fragments and reads.
   PlasmidBase   - Class in  bioroot.plasmid      PlasmidBase()   - 
Constructor for class bioroot.plasmid. PlasmidBase 
  
   PlasmidBean   - Class in  bioroot.plasmid      PlasmidBean()   - 
Constructor for class bioroot.plasmid. PlasmidBean 
  
   PlasmidBean(int, DBUtil)   - 
Constructor for class bioroot.plasmid. PlasmidBean 
  
   PlasmidBean(ResultSet)   - 
Constructor for class bioroot.plasmid. PlasmidBean 
  
   PlasmidBean(String[], IdNameMatch[], IdNameMatch[], IdNameMatch[], UserBean, DBUtil)   - 
Constructor for class bioroot.plasmid. PlasmidBean 
 For bulk file upload should be 16 cells 
         0      1          2        3      4           5               6                 7              8            9       10            11        12      13     14  15                                                      16                              
         Name Alias Source Recipients Reference Gene Organism Markers StrainIds Location Concentration Volume Purification Availability VisibilityBean Notes Sequence
   PlasmidModify   - Class in  bioroot.plasmid  Servlet to modify plasmids using PlasmidBeans referenced from a Session object.   PlasmidModify()   - 
Constructor for class bioroot.plasmid. PlasmidModify 
  
   PlasmidSpreadSheet   - Class in  bioroot.plasmid      PlasmidSpreadSheet()   - 
Constructor for class bioroot.plasmid. PlasmidSpreadSheet 
  
   PlasmidUpload   - Class in  bioroot.plasmid      PlasmidUpload()   - 
Constructor for class bioroot.plasmid. PlasmidUpload 
  
   PlasmidUploadBean   - Class in  bioroot.plasmid  Plasmid bulk file upload bean.   PlasmidUploadBean()   - 
Constructor for class bioroot.plasmid. PlasmidUploadBean 
  
   PlasmidViewPreferences   - Class in  bioroot.plasmid      PlasmidViewPreferences()   - 
Constructor for class bioroot.plasmid. PlasmidViewPreferences 
  
   plot()   - 
Method in class trans.graphics. AggregatePlots 
  
   PlotFloatArrays   - Class in  trans.graphics      PlotFloatArrays()   - 
Constructor for class trans.graphics. PlotFloatArrays 
  
   plotHistogram()   - 
Method in class selex. SelexParams 
  
   PlotterPreferences   - Class in  gata.main      PlotterPreferences()   - 
Constructor for class gata.main. PlotterPreferences 
  
   pmX   - 
Variable in class trans.tpmap. MapFeature 
  
   pmY   - 
Variable in class trans.tpmap. MapFeature 
  
   Point   - Class in  edu.utah.seq.data      Point(int, float)   - 
Constructor for class edu.utah.seq.data. Point 
  
   PointData   - Class in  edu.utah.seq.data  Container for holding genomic positions - scores data.   PointData()   - 
Constructor for class edu.utah.seq.data. PointData 
  
   PointData(File, boolean)   - 
Constructor for class edu.utah.seq.data. PointData 
 Reads in a barFile creating a PointData object.
   poissonProbabilityApproximation(double, int)   - 
Static method in class util.gen. Num 
 Calculates a natural log approximation of the Poisson probablility.
   poissonProbablility(double, int)   - 
Static method in class util.gen. Num 
 Calculates the Poisson probablility for observed    poissonPValues(int, double, File, File, double)   - 
Static method in class util.gen. Num 
 Calculates p-values using a Poisson probablility from built in R function.
   pooledSubArray(float[][], float[][], int, int)   - 
Static method in class util.gen. Num 
 Builds a pooled from part of a float[replica][intensities] for treat and control.
   populateBean(Object, HttpServletRequest)   - 
Static method in class util.gen. HTML 
 Fills in all the params of a bean.
   position   - 
Variable in class trans.cel. Quantile 
  
   positive(Positive[], Sgr)   - 
Static method in class trans.roc. MethodTester 
 Checks to see if an sgr object is one of the positives, if not returns -1, otherwise returns index number.
   Positive   - Class in  trans.roc  Container for a positive BAC used by the Sgr scripts.   Positive(String, int, int)   - 
Constructor for class trans.roc. Positive 
  
   positive(Positive[], Sgr)   - 
Static method in class trans.roc. SgrBacCounter 
 Checks to see if an sgr object is one of the positives, if not returns -1, otherwise returns index number.
   PositiveComparator   - Class in  trans.roc      PositiveComparator()   - 
Constructor for class trans.roc. PositiveComparator 
  
   PosRegion   - Class in  trans.main  For holding information about a genomic interval, what genes are up, down, overlap, or contain this genomic inteval.   PosRegion(String, String, int, int)   - 
Constructor for class trans.main. PosRegion 
  
   PossumToGFF   - Class in  util.bio.converters  Converts a copied Possum output to GFF annotation, prints to screen, sorted by score.   PossumToGFF()   - 
Constructor for class util.bio.converters. PossumToGFF 
  
   postMail(String[], String, String, String)   - 
Method in class util.gen. Email 
  
   postMailNoAuthentication(String[], String, String, String, String, String)   - 
Static method in class util.gen. Email 
 Simple static method for sending email, no authentication, modified from http://www.javacommerce.com/articles/sendingmail.htm Sudhir Ancha.
   postWithAttachment(String[], String, String, String, String, String)   - 
Method in class util.gen. Email 
  
   Preference   - Class in  bioroot      Preference()   - 
Constructor for class bioroot. Preference 
  
   Preference(String)   - 
Constructor for class bioroot. Preference 
 databaseConcat = visibility : orderBy: labGroups : features separated by colons,
        labGroups and feature lines separated by semi-colons
   PrependColumn   - Class in  trans.misc      PrependColumn()   - 
Constructor for class trans.misc. PrependColumn 
  
   prependDouble(double[], double)   - 
Static method in class util.gen. Num 
 Returns a new array with the newValue appended on the beginning.
   prependString(String[], String)   - 
Static method in class util.gen. Misc 
 Returns a new array with the newValue appended on the beginning.
   prependTargetMedianInControlStats()   - 
Method in class trans.cel. QuantileNormalization 
  
   Primer3Pick   - Class in  util.bio.wrappers  Container for sequence used in picking primers with Primer3.   Primer3Pick(String)   - 
Constructor for class util.bio.wrappers. Primer3Pick 
  
   Primer3Wrapper   - Class in  util.bio.wrappers  This app wraps the command line primer3 app to make it useful for picking qPCR primers.   Primer3Wrapper(String[])   - 
Constructor for class util.bio.wrappers. Primer3Wrapper 
 Uses a shell script file to execute the primer3 app due to a problem feeding to STDIN from java.
   print(String, boolean[], PrintWriter)   - 
Static method in class trans.anno. MergeRegions 
  
   printAlignment()   - 
Method in class gata.aligner. Alignment 
  
   printAllExons(int)   - 
Method in class util.bio.parsers. UCSCGeneModelTableReader 
 Prints all exons to standard out.
   printArray(float[][])   - 
Static method in class trans.graphics. AggregatePlots 
 Prints x as row, y as column with row index, float[y][x]
   printArray(Object[])   - 
Static method in class util.gen. Misc 
 Prints a Object[] to System.out
   printArray(byte[])   - 
Static method in class util.gen. Misc 
 Prints a byte[] to System.out
   printArray(boolean[])   - 
Static method in class util.gen. Misc 
 Prints a boolean[] to System.out
   printArray(Object[][])   - 
Static method in class util.gen. Misc 
 Prints a Object[][] to System.out
   printArray(String[])   - 
Static method in class util.gen. Misc 
 Prints a String[] to System.out
   printArray(ArrayList)   - 
Static method in class util.gen. Misc 
 Prints ArrayList to System.out
   printArray(int[])   - 
Static method in class util.gen. Misc 
 Prints a int[] to System.out
   printArray(float[])   - 
Static method in class util.gen. Misc 
 Prints a int[] to System.out
   printArray(double[])   - 
Static method in class util.gen. Misc 
 Prints a double[] to System.out on a single line.
   printArray(double[][])   - 
Static method in class util.gen. Misc 
 Print to System.out a double[][]
   printArray(float[][])   - 
Static method in class util.gen. Misc 
 Print to System.out a float[][]
   printArray(int[][])   - 
Static method in class util.gen. Misc 
 Print to System.out a int[][]
   printArrayLine(String[])   - 
Static method in class util.gen. Misc 
 Prints a String[] to System.out
   printBarFiles()   - 
Method in class expr. CorrelationMaps 
  
   printBinarySequence(File, File)   - 
Method in class edu.utah.seq.compression. CompressFasta 
  
   printBins()   - 
Method in class util.gen. Histogram 
 Prints bin info including mean if scores were provided while counting.
   printBlocks(int[][], String, File)   - 
Method in class util.bio.annotation. ExportIntergenicRegions 
 Prints a file containing the block regions.
   printCalculations(ArrayList, int)   - 
Static method in class trans.misc. CalcGCAndTransform 
 Prints %GC, Mean, 3xStdErr
   printCalculations(ArrayList, int)   - 
Static method in class trans.misc. CalcGCRatiosFromIntervals 
 Prints %GC, Mean, 3xStdErr
   printCelFileStats()   - 
Method in class trans.qc. CelFileQualityControl 
 Runs through groups of cel files, calling statSingleCelFile() on each
 and prints the results.
   printCenteredForwardAndReverseCounts()   - 
Method in class util.gen. Histogram 
 Prints two columns, forward counts and reversed counts centered on max value with zeros as filler.
   printChroms(RocWindow[])   - 
Static method in class trans.roc. ParsePatternedWindows 
  
   printClusters(LinkedHashMap)   - 
Method in class util.bio.parsers.gff. ExtractGffFeatures 
 Prints all the gff lines.
   printCoorGenes(FindNeighboringGenes.CoorGene[])   - 
Method in class trans.anno. FindNeighboringGenes 
  
   printCorrelationWindows()   - 
Method in class expr. CorrelationMaps 
 Prints a spread sheet summary and a xxx.egr file for all of the windows.
   printDistToClosestATGAndTranscript(BindingRegion[])   - 
Method in class trans.anno. AnnotateRegions 
 Prints rank, chrom, start, stop, distance to closest ATG, to closest transcript start.
   printDistToClosestATGAndTranscript(BindingRegion[])   - 
Method in class trans.anno. AnnotateRegionsWithGeneList 
 Prints rank, chrom, start, stop, distance to closest ATG, to closest transcript start.
   printDoc()   - 
Static method in class selex. UtilSelex 
  
   printDocs()   - 
Static method in class edu.utah.seq.analysis. DefinedRegionScanSeqs 
  
   printDocs()   - 
Static method in class edu.utah.seq.analysis. EnrichedRegionMaker 
  
   printDocs()   - 
Static method in class edu.utah.seq.analysis. QCSeqs 
  
   printDocs()   - 
Static method in class edu.utah.seq.analysis. ScanSeqs 
  
   printDocs()   - 
Static method in class edu.utah.seq.data. FilterPointData 
  
   printDocs()   - 
Static method in class edu.utah.seq.data. SubSamplePointData 
  
   printDocs()   - 
Static method in class edu.utah.seq.parsers. ElandMultiParser 
  
   printDocs()   - 
Static method in class edu.utah.seq.parsers. ElandParser 
  
   printDocs()   - 
Static method in class edu.utah.seq.parsers. ElandSequenceParser 
  
   printDocs()   - 
Static method in class edu.utah.seq.parsers. Tag2Point 
  
   printDocs()   - 
Static method in class expr. CorrelationMaps 
  
   printDocs()   - 
Static method in class expr. FileCrossFilter 
  
   printDocs()   - 
Static method in class expr. FileMatchJoiner 
  
   printDocs()   - 
Static method in class expr. ParseAgilentData 
  
   printDocs()   - 
Static method in class igb.util. Windows2HeatMapSgr 
  
   printDocs()   - 
Static method in class meme. UtilMeme 
  
   printDocs()   - 
Static method in class trans.anno. AnnotateRegions 
  
   printDocs()   - 
Static method in class trans.anno. AnnotateRegionsWithGeneList 
  
   printDocs()   - 
Static method in class trans.anno. FindNeighboringGenes 
  
   printDocs()   - 
Static method in class trans.anno. IntersectKeyWithRegions 
  
   printDocs()   - 
Static method in class trans.anno. IntersectRegions 
  
   printDocs()   - 
Static method in class trans.cel. CelFileConverter 
  
   printDocs()   - 
Static method in class trans.cel. CelProcessor 
  
   printDocs()   - 
Static method in class trans.cel. MakeChromosomeSets 
  
   printDocs()   - 
Static method in class trans.graphics. AggregatePlots 
  
   printDocs()   - 
Static method in class trans.graphics. CelMasker 
  
   printDocs()   - 
Static method in class trans.graphics. IntervalPlotter 
  
   printDocs()   - 
Static method in class trans.graphics. RankedSetAnalysis 
  
   printDocs()   - 
Static method in class trans.graphics. VirtualCel 
  
   printDocs()   - 
Static method in class trans.main. BindingRegionGraphPrinter 
  
   printDocs()   - 
Static method in class trans.main. ExportIntervalData 
  
   printDocs()   - 
Static method in class trans.main. FDRWindowConverter 
  
   printDocs()   - 
Static method in class trans.main. FindSubBindingRegions 
  
   printDocs()   - 
Static method in class trans.main. IntensityPrinter 
  
   printDocs()   - 
Static method in class trans.main. IntervalFilter 
  
   printDocs()   - 
Static method in class trans.main. IntervalGFFPrinter 
  
   printDocs()   - 
Static method in class trans.main. IntervalGraphPrinter 
  
   printDocs()   - 
Static method in class trans.main. IntervalMaker 
  
   printDocs()   - 
Static method in class trans.main. IntervalReportPrinter 
  
   printDocs()   - 
Static method in class trans.main. LoadChipSetIntervalOligoInfo 
  
   printDocs()   - 
Static method in class trans.main. LoadIntervalOligoInfo 
  
   printDocs()   - 
Static method in class trans.main. MakeAllWindows 
  
   printDocs()   - 
Static method in class trans.main. MergeWindowArrays 
  
   printDocs()   - 
Static method in class trans.main. MultiSGRPrinter 
  
   printDocs()   - 
Static method in class trans.main. MultiWindowIntervalMaker 
  
   printDocs()   - 
Static method in class trans.main. OligoIntensityPrinter 
  
   printDocs()   - 
Static method in class trans.main. OverlapCounter 
  
   printDocs()   - 
Static method in class trans.main. ScanChip 
  
   printDocs()   - 
Static method in class trans.main. ScanChromosomes 
  
   printDocs()   - 
Static method in class trans.main. ScanChromosomesCNV 
  
   printDocs()   - 
Static method in class trans.main. ScanGenes 
  
   printDocs()   - 
Static method in class trans.main. ScoreIntervals 
  
   printDocs()   - 
Static method in class trans.main. SetNumberIntervalMaker 
  
   printDocs()   - 
Static method in class trans.main. SplitWindowArraysByChromosome 
  
   printDocs()   - 
Static method in class trans.main. T2 
  
   printDocs()   - 
Static method in class trans.main. TestScanChip 
  
   printDocs()   - 
Static method in class trans.main. WindowBlockMaker 
  
   printDocs()   - 
Static method in class trans.main. WindowBlockMakerTwoColor 
  
   printDocs()   - 
Static method in class trans.misc. Bar2Gr 
  
   printDocs()   - 
Static method in class trans.misc. BestWindowScoreExtractor 
  
   printDocs()   - 
Static method in class trans.misc. CalculateGeneExtensions 
  
   printDocs()   - 
Static method in class trans.misc. ConvertAgilentData 
  
   printDocs()   - 
Static method in class trans.misc. ConvertBars2TPMapCel 
  
   printDocs()   - 
Static method in class trans.misc. ConvertGeoData 
  
   printDocs()   - 
Static method in class trans.misc. ConvertNimblegenNDF2TPMap 
  
   printDocs()   - 
Static method in class trans.misc. ConvertNimblegenPAIR2Cela 
  
   printDocs()   - 
Static method in class trans.misc. Gr2Bar 
  
   printDocs()   - 
Static method in class trans.misc. Sgr2Bar 
  
   printDocs()   - 
Static method in class trans.misc. Wig2Bar 
  
   printDocs()   - 
Static method in class trans.qc. CelFileQualityControl 
  
   printDocs()   - 
Static method in class trans.qc. CoordinateExtractor1lq 
  
   printDocs()   - 
Static method in class trans.qc. Parser1lq 
  
   printDocs()   - 
Static method in class trans.roc. ParsePatternedWindows 
  
   printDocs()   - 
Static method in class trans.roc. PWMScoreWindows 
  
   printDocs()   - 
Static method in class trans.roc. RocWindowScanner 
  
   printDocs()   - 
Static method in class trans.roc. ScoreParsedBars 
  
   printDocs()   - 
Static method in class trans.tpmap. ConvertTPMapsToFeatures 
  
   printDocs()   - 
Static method in class trans.tpmap. FilterTPMapByRegions 
  
   printDocs()   - 
Static method in class trans.tpmap. MummerMapper 
  
   printDocs()   - 
Static method in class trans.tpmap. OligoFlipper 
  
   printDocs()   - 
Static method in class trans.tpmap. TPMapDensityReducer 
  
   printDocs()   - 
Static method in class trans.tpmap. TPMapOligoBlastFilter 
  
   printDocs()   - 
Static method in class trans.tpmap. TPMapProcessor 
  
   printDocs()   - 
Static method in class trans.tpmap. TPMapSort 
  
   printDocs()   - 
Static method in class util.apps. Correlate 
  
   printDocs()   - 
Static method in class util.apps. FileJoiner 
  
   printDocs()   - 
Static method in class util.apps. FileSplitter 
  
   printDocs()   - 
Static method in class util.apps. FileTrimmer 
  
   printDocs()   - 
Static method in class util.apps.gump. Gump 
  
   printDocs()   - 
Static method in class util.apps. IntersectLists 
  
   printDocs()   - 
Static method in class util.apps. JQSub 
  
   printDocs()   - 
Static method in class util.apps. PrintSelectColumns 
  
   printDocs()   - 
Static method in class util.apps. ScatterPlot 
  
   printDocs()   - 
Static method in class util.apps. ScoreChromosomes 
  
   printDocs()   - 
Static method in class util.apps. ScoreSequences 
  
   printDocs()   - 
Static method in class util.bio.annotation. ExportIntergenicRegions 
  
   printDocs()   - 
Static method in class util.bio.annotation. ExportIntronicRegions 
  
   printDocs()   - 
Static method in class util.bio.cluster. HierarchicalClustering 
  
   printDocs()   - 
Static method in class util.bio.parsers. FetchGenomicSequences 
  
   printDocs()   - 
Static method in class util.bio.parsers.gff. ExtractGffFeatures 
  
   printDocs()   - 
Static method in class util.bio.parsers.gff. ParseWormBaseGffToUCSC 
  
   printDocs()   - 
Static method in class util.bio.parsers. SynonymMatching 
  
   printDocs()   - 
Static method in class util.bio.seq. ConvertFasta2GCBoolean 
  
   printDocs()   - 
Static method in class util.bio.seq. ConvertFasta2RealSeqBoolean 
  
   printDocs()   - 
Static method in class util.bio.seq. IndexFastas 
  
   printDocs()   - 
Static method in class util.bio.seq. MakeSpliceJunctionFasta 
  
   printDocs()   - 
Static method in class util.bio.seq. MakeTranscriptome 
  
   printDocs()   - 
Static method in class util.bio.seq. OligoTiler 
  
   printDocs()   - 
Static method in class util.bio.wrappers. Primer3Wrapper 
  
   printEachArray(float[][])   - 
Static method in class trans.graphics. AggregatePlots 
 Prints x as row, y as column with row index, float[y][x]
   printEGRFile(EnrichedRegion[], File, String, String[])   - 
Static method in class edu.utah.seq.analysis. EnrichedRegionMaker 
 Writes out an egr file, used by IGB for graph display.
   printEGRFileForBestSubWindow(EnrichedRegion[], File, String)   - 
Static method in class edu.utah.seq.analysis. EnrichedRegionMaker 
 Writes out an egr file for best sub windows, used by IGB for graph display.
   printEnrichedRegionReport(EnrichedRegion[], File, String, String[])   - 
Static method in class edu.utah.seq.analysis. EnrichedRegionMaker 
 Writes out an excel compatible tab delimited spreadsheet with a hyperlink to IGB.
   printEnz()   - 
Method in class util.bio.digest. Enzyme 
  
   printErrAndExit(String)   - 
Static method in class util.gen. Misc 
 Prints message to screen, then exits.
   printExit(String)   - 
Static method in class util.gen. Misc 
 Prints message to screen, then exits.
   printFasta()   - 
Method in class util.bio.parsers. MultiFastaParser 
  
   printForGeoCode()   - 
Method in class util.apps. ParseMLSHTML 
  
   printGeneModels()   - 
Method in class edu.utah.seq.analysis. DefinedRegionScanSeqs 
  
   printGFF()   - 
Method in class trans.main. IntervalGFFPrinter 
  
   printGFFFile(EnrichedRegion[], File, String, int, String[])   - 
Static method in class edu.utah.seq.analysis. EnrichedRegionMaker 
 Prints a simple gff file, the index is used to pick a score to place in the score column.
   printGFFFileForBestSubWindow(EnrichedRegion[], File, String)   - 
Static method in class edu.utah.seq.analysis. EnrichedRegionMaker 
 Prints a simple gff file, the index is used to pick a score to place in the score column.
   printHashMap(HashMap, String)   - 
Static method in class util.gen. Misc 
 Prints key + seperator + value+ return to out.
   printHashSetToFile(HashSet, String)   - 
Static method in class util.gen. Misc 
 Prints a HashSet to a file.
   printHistogram(int[], int[], String[])   - 
Static method in class util.gen. Histogram 
 Prints a simple histogram scaled using stars but real counts shown.
   printHistogram(int[], PrintWriter)   - 
Static method in class util.gen. Histogram 
 Prints a simple histogram to a PrintWriter.
   printHistogram(int[])   - 
Static method in class util.gen. Histogram 
 Prints a simple histogram.
   printIntensityStats()   - 
Method in class trans.cel. QuantileNormalization 
 Prints statistics about the current intensity float[][] arrays
   printIntervalGraphs()   - 
Method in class trans.main. T2 
 Prints interval bed and sgr files via IntervalGraphPrinter.
   printIt()   - 
Method in class edu.utah.seq.methylation454. ReadAlignParser 
  
   printLocalAlignment()   - 
Method in class gata.aligner. LocalAlignment 
  
   printMFP()   - 
Method in class util.bio.parsers. QualityFileParser 
  
   printNotes()   - 
Method in class util.bio.parsers.gff. Gff3Parser 
  
   printOligoRatios()   - 
Method in class trans.main. ScanChip 
 Prints log2 oligo ratios.
   printOligoRatios()   - 
Method in class trans.main. ScanChromosomes 
 Prints log2 oligo ratios as a chromosome specific xxx.bar file.
   printOligoRatios()   - 
Method in class trans.main. ScanChromosomesCNV 
 Prints log2 oligo ratios as a chromosome specific xxx.bar file.
   printOutMap()   - 
Method in class edu.utah.seq.parsers. ElandMultiParser 
  
   printRankedIntersection(int[])   - 
Method in class trans.graphics. RankedSetAnalysis 
 Prints to screen the results of rankedIntersection(GenomicRegion[] r).
   printRankedIntersectionAll(GenomicRegion[])   - 
Method in class trans.graphics. RankedSetAnalysis 
 Rank analysis where each regions is check against the entire other list.
   printRatios(Interval)   - 
Method in class trans.main. IntervalReportPrinter 
 Used when making mock Intervals from which you want to calculate various oligo based measurements.
   printReport()   - 
Method in class trans.main. IntervalReportPrinter 
  
   printReport()   - 
Method in class util.bio.parsers. QualityFileParser 
  
   printResults()   - 
Method in class util.apps. GeneCentricCNVParser 
  
   printSave(String)   - 
Method in class meme. MemeResults 
  
   printSave(String)   - 
Method in class selex. SelexParams 
  
   printSaveIntervalScoreGraphFiles()   - 
Method in class trans.main. IntervalGraphPrinter 
 Prints and saves a Affy .sgr file
   printScaledHistogram()   - 
Method in class util.gen. Histogram 
 Prints scaled histogram.
   printScores(Interval[], File, boolean)   - 
Static method in class trans.main. IntervalGraphPrinter 
 Prints to a File a line for every Oligo in each Interval.
   PrintSelectColumns   - Class in  util.apps  Prints out select columns from a tab delimited file, skips lines, appends words, etc.   PrintSelectColumns(String[])   - 
Constructor for class util.apps. PrintSelectColumns 
  
   printSeqFiles()   - 
Method in class selex. SeqFiles 
  
   printSeqRead()   - 
Method in class selex. SeqRead 
  
   printSeqsToConsole()   - 
Method in class gata.plotter. ConservedSeqs 
  
   printSgrLines()   - 
Method in class trans.main. WindowBlockMakerTwoColor 
  
   printSGRWindows(RocWindow[])   - 
Static method in class trans.roc. RocWindow 
  
   printSpreadSheets()   - 
Method in class trans.main. T2 
 Prints interval reports in a spread sheet via IntervalReportPrinter.
   printStatFlags()   - 
Method in class trans.qc. CelFileQualityControl 
 Prints name, median min max for each stat flag
   printStringArray(String[], String)   - 
Static method in class util.bio.converters. RepeatMaskerToGFF 
 Prints out the string array on a line divided by the separator, good for debugging
   printSubSeq()   - 
Method in class selex. SubSeq 
  
   printSubWindowGraphFiles(EnrichedRegion[], File, String)   - 
Method in class edu.utah.seq.analysis. EnrichedRegionMaker 
  
   printTable(Object[][], String[])   - 
Static method in class trans.main. OverlapCounter 
  
   printToTextArea(String)   - 
Method in class gata.plotter. Console 
  
   printToTextArea(String)   - 
Method in class util.apps.gump. GumpResults 
  
   printTruncatedSeqsToConsole(int, int, int, int)   - 
Method in class gata.plotter. ConservedSeqs 
  
   printUCSCFormat(LinkedHashMap)   - 
Method in class util.bio.parsers.gff. ExtractGffFeatures 
 Print each cluster in ucsc table format
                refGene.name    refGene.chrom   refGene.strand  refGene.txStart refGene.txEnd   refGene.cdsStart        refGene.cdsEnd  refGene.exonCount       refGene.exonStarts      refGene.exonEnds        refLink.product geneName.name   refSeqSummary.summary
                 NM_198576      chr1    +       995569  1031415 995619  1030284 36      995569,997647,1010723,1016111,1016522,1016827,1017258,1018541,1018840,1019125,1019411,1019636,1020463,1020661,1021035,1021266,1021462,1021699,1022122,1022629,1022875,1023078,1023314,1024169,1024538,1024868,1025205,1025535,1025729,1026028,1026555,1026755,1027030,1029055,1029750,1030126,  995820,997909,1010771,1016327,1016747,1017052,1017465,1018760,1019035,1019326,1019560,1019742,1020580,1020826,1021179,1021391,1021568,1022038,1022260,1022757,1022990,1023198,1023668,1024362,1024754,1025098,1025340,1025632,1025894,1026140,1026672,1026948,1027118,1029280,1029854,1031415,  agrin   AGRIN   Agrin is a neuronal aggregating factor that induces the aggregation of ...
   printUCSCFormat(File)   - 
Method in class util.bio.parsers.gff. ParseWormBaseGffToUCSC 
 Print each cluster in ucsc table format
                refGene.common name refGene.name refGene.chrom refGene.strand refGene.txStart refGene.txEnd refGene.cdsStart refGene.cdsEnd     refGene.exonCount       refGene.exonStarts      refGene.exonEnds
                 p53 NM_198576  chr1    +       995569  1031415 995619  1030284 36      995569,997647,1010723,1016111,1016522,1016827,1017258,1018541,1018840,1019125,1019411,1019636,1020463,1020661,1021035,1021266,1021462,1021699,1022122,1022629,1022875,1023078,1023314,1024169,1024538,1024868,1025205,1025535,1025729,1026028,1026555,1026755,1027030,1029055,1029750,1030126,  995820,997909,1010771,1016327,1016747,1017052,1017465,1018760,1019035,1019326,1019560,1019742,1020580,1020826,1021179,1021391,1021568,1022038,1022260,1022757,1022990,1023198,1023668,1024362,1024754,1025098,1025340,1025632,1025894,1026140,1026672,1026948,1027118,1029280,1029854,1031415,  agrin   AGRIN   Agrin is a neuronal aggregating factor that induces the aggregation of ...
   printUCSCTableFormat(HashMap)   - 
Method in class util.bio.parsers. RefSeqAnnotationParser 
  
   processArgs(String[])   - 
Method in class edu.utah.seq.analysis. DefinedRegionScanSeqs 
 This method will process each argument and assign new varibles
   processArgs(String[])   - 
Method in class edu.utah.seq.analysis. EnrichedRegionMaker 
 This method will process each argument and assign new varibles
   processArgs(String[])   - 
Method in class edu.utah.seq.analysis. QCSeqs 
 This method will process each argument and assign new varibles
   processArgs(String[])   - 
Method in class edu.utah.seq.analysis. ScanSeqs 
 This method will process each argument and assign new varibles
   processArgs(String[])   - 
Method in class edu.utah.seq.data. FilterPointData 
 This method will process each argument and assign new varibles
   processArgs(String[])   - 
Method in class edu.utah.seq.data. SubSamplePointData 
 This method will process each argument and assign new varibles
   processArgs(String[])   - 
Method in class edu.utah.seq.parsers. ElandMultiParser 
 This method will process each argument and assign new varibles
   processArgs(String[])   - 
Method in class edu.utah.seq.parsers. ElandParser 
 This method will process each argument and assign new varibles
   processArgs(String[])   - 
Method in class edu.utah.seq.parsers. ElandSequenceParser 
 This method will process each argument and assign new varibles
   processArgs(String[])   - 
Method in class edu.utah.seq.parsers. Tag2Point 
 This method will process each argument and assign new varibles
   processArgs(String[])   - 
Method in class expr. CorrelationMaps 
 This method will process each argument and assign new varibles
   processArgs(String[])   - 
Method in class expr. FileCrossFilter 
 This method will process each argument and assign new varibles
   processArgs(String[])   - 
Method in class expr. FileMatchJoiner 
 This method will process each argument and assign new varibles
   processArgs(String[])   - 
Method in class expr. ParseAgilentData 
 This method will process each argument and assign new varibles
   processArgs(String[])   - 
Method in class igb.util. Windows2HeatMapSgr 
 This method will process each argument and assign any new varibles
   processArgs(String[])   - 
Method in class selex. SelexParams 
  
   processArgs(String[])   - 
Method in class trans.anno. AnnotateRegions 
 This method will process each argument and assign new varibles
   processArgs(String[])   - 
Method in class trans.anno. AnnotateRegionsWithGeneList 
 This method will process each argument and assign new varibles
   processArgs(String[])   - 
Method in class trans.anno. FindNeighboringGenes 
 This method will process each argument and assign new varibles
   processArgs(String[])   - 
Method in class trans.anno. IntersectKeyWithRegions 
 This method will process each argument and assign new varibles
   processArgs(String[])   - 
Method in class trans.anno. IntersectRegions 
 This method will process each argument and assign new varibles
   processArgs(String[])   - 
Method in class trans.cel. CelFileConverter 
 This method will process each argument and assign any new varibles
   processArgs(String[])   - 
Method in class trans.cel. CelProcessor 
 This method will process each argument and assign any new varibles
   processArgs(String[])   - 
Method in class trans.cel. MakeChromosomeSets 
 This method will process each argument and assign any new varibles
   processArgs(String[])   - 
Method in class trans.graphics. AggregatePlots 
 This method will process each argument and assign new varibles
   processArgs(String[])   - 
Method in class trans.graphics. CelMasker 
 This method will process each argument and assign any new varibles
   processArgs(String[])   - 
Method in class trans.graphics. IntervalPlotter 
 This method will process each argument and assign any new varibles
   processArgs(String[])   - 
Method in class trans.graphics. RankedSetAnalysis 
 This method will process each argument and assign any new varibles
   processArgs(String[])   - 
Method in class trans.graphics. VirtualCel 
 This method will process each argument and assign any new varibles
   processArgs(String[])   - 
Method in class trans.main. BindingRegionGraphPrinter 
 This method will process each argument and assign any new varibles
   processArgs(String[])   - 
Method in class trans.main. ExportIntervalData 
 This method will process each argument and assign new varibles
   processArgs(String[])   - 
Method in class trans.main. FDRWindowConverter 
 This method will process each argument and assign new varibles
   processArgs(String[])   - 
Method in class trans.main. FindSubBindingRegions 
 This method will process each argument and assign new varibles
   processArgs(String[])   - 
Method in class trans.main. IntensityPrinter 
 This method will process each argument and assign any new varibles
   processArgs(String[])   - 
Method in class trans.main. IntervalFilter 
 This method will process each argument and assign any new varibles
   processArgs(String[])   - 
Method in class trans.main. IntervalGFFPrinter 
 This method will process each argument and assign any new varibles
   processArgs(String[])   - 
Method in class trans.main. IntervalGraphPrinter 
 This method will process each argument and assign any new varibles
   processArgs(String[])   - 
Method in class trans.main. IntervalMaker 
 This method will process each argument and assign new varibles
   processArgs(String[])   - 
Method in class trans.main. IntervalReportPrinter 
 This method will process each argument and assign any new varibles
   processArgs(String[])   - 
Method in class trans.main. LoadChipSetIntervalOligoInfo 
 This method will process each argument and assign new varibles
   processArgs(String[])   - 
Method in class trans.main. LoadIntervalOligoInfo 
 This method will process each argument and assign new fields
   processArgs(String[])   - 
Method in class trans.main. MakeAllWindows 
 This method will process each argument and assign new varibles
   processArgs(String[])   - 
Method in class trans.main. MergeWindowArrays 
 This method will process each argument and assign new varibles
   processArgs(String[])   - 
Method in class trans.main. MultiSGRPrinter 
 This method will process each argument and assign new varibles
   processArgs(String[])   - 
Method in class trans.main. MultiWindowIntervalMaker 
 This method will process each argument and assign new varibles
   processArgs(String[])   - 
Method in class trans.main. OligoIntensityPrinter 
 This method will process each argument and assign any new varibles
   processArgs(String[])   - 
Method in class trans.main. OverlapCounter 
 This method will process each argument and assign any new varibles
   processArgs(String[])   - 
Method in class trans.main. ScanChip 
 This method will process each argument and assign new varibles
   processArgs(String[])   - 
Method in class trans.main. ScanChromosomes 
 This method will process each argument and assign new varibles
   processArgs(String[])   - 
Method in class trans.main. ScanChromosomesCNV 
 This method will process each argument and assign new varibles
   processArgs(String[])   - 
Method in class trans.main. ScanGenes 
 This method will process each argument and assign new varibles
   processArgs(String[])   - 
Method in class trans.main. ScoreIntervals 
 This method will process each argument and assign any new varibles
   processArgs(String[])   - 
Method in class trans.main. SetNumberIntervalMaker 
 This method will process each argument and assign new varibles
   processArgs(String[])   - 
Method in class trans.main. SplitWindowArraysByChromosome 
 This method will process each argument and assign new varibles
   processArgs(String[])   - 
Method in class trans.main. T2 
 This method will process each argument and assign new varibles
   processArgs(String[])   - 
Method in class trans.main. TestScanChip 
 This method will process each argument and assign new varibles
   processArgs(String[])   - 
Method in class trans.main. WindowBlockMaker 
 This method will process each argument and assign new varibles
   processArgs(String[])   - 
Method in class trans.main. WindowBlockMakerTwoColor 
 This method will process each argument and assign new varibles
   processArgs(String[])   - 
Method in class trans.misc. Bar2Gr 
 This method will process each argument and assign new varibles
   processArgs(String[])   - 
Method in class trans.misc. BestWindowScoreExtractor 
 This method will process each argument and assign any new varibles
   processArgs(String[])   - 
Method in class trans.misc. CalculateGeneExtensions 
 This method will process each argument and assign new varibles
   processArgs(String[])   - 
Method in class trans.misc. ConvertAgilentData 
 This method will process each argument and assign new varibles
   processArgs(String[])   - 
Method in class trans.misc. ConvertBars2TPMapCel 
 This method will process each argument and assign new variables
   processArgs(String[])   - 
Method in class trans.misc. ConvertGeoData 
 This method will process each argument and assign new varibles
   processArgs(String[])   - 
Method in class trans.misc. ConvertNimblegenNDF2TPMap 
 This method will process each argument and assign new varibles
   processArgs(String[])   - 
Method in class trans.misc. ConvertNimblegenPAIR2Cela 
 This method will process each argument and assign new varibles
   processArgs(String[])   - 
Method in class trans.misc. Gr2Bar 
 This method will process each argument and assign new varibles
   processArgs(String[])   - 
Method in class trans.misc. Sgr2Bar 
 This method will process each argument and assign new varibles
   processArgs(String[])   - 
Method in class trans.misc. Wig2Bar 
 This method will process each argument and assign new varibles
   processArgs(String[])   - 
Method in class trans.qc. CelFileQualityControl 
 This method will process each argument and assign any new varibles
   processArgs(String[])   - 
Method in class trans.qc. CoordinateExtractor1lq 
 This method will process each argument and assign any new varibles
   processArgs(String[])   - 
Method in class trans.qc. Parser1lq 
 This method will process each argument and assign any new varibles
   processArgs(String[])   - 
Method in class trans.roc. ParsePatternedWindows 
 This method will process each argument and assign new varibles
   processArgs(String[])   - 
Method in class trans.roc. PWMScoreWindows 
 This method will process each argument and assign new varibles
   processArgs(String[])   - 
Method in class trans.roc. RocWindowScanner 
 This method will process each argument and assign new varibles
   processArgs(String[])   - 
Method in class trans.roc. ScoreParsedBars 
 This method will process each argument and assign new varibles
   processArgs(String[])   - 
Method in class trans.tpmap. ConvertTPMapsToFeatures 
 This method will process each argument and assign any new varibles
   processArgs(String[])   - 
Method in class trans.tpmap. FilterTPMapByRegions 
 This method will process each argument and assign any new varibles
   processArgs(String[])   - 
Method in class trans.tpmap. MummerMapper 
 This method will process each argument and assign any new varibles
   processArgs(String[])   - 
Method in class trans.tpmap. OligoFlipper 
 This method will process each argument and assign any new varibles
   processArgs(String[])   - 
Method in class trans.tpmap. TPMapDensityReducer 
 This method will process each argument and assign any new varibles
   processArgs(String[])   - 
Method in class trans.tpmap. TPMapOligoBlastFilter 
 This method will process each argument and assign any new varibles
   processArgs(String[])   - 
Method in class trans.tpmap. TPMapProcessor 
 This method will process each argument and assign any new varibles
   processArgs(String[])   - 
Method in class trans.tpmap. TPMapSort 
 This method will process each argument and assign any new varibles
   processArgs(String[])   - 
Method in class util.apps. Correlate 
 This method will process each argument and assign new varibles
   processArgs(String[])   - 
Method in class util.apps. FileJoiner 
 This method will process each argument and assign any new varibles
   processArgs(String[])   - 
Method in class util.apps. FileSplitter 
 This method will process each argument and assign any new varibles
   processArgs(String[])   - 
Method in class util.apps. FileTrimmer 
 This method will process each argument and assign any new varibles
   processArgs(String[])   - 
Method in class util.apps.gump. Gump 
 This method will process each argument and assign new varibles
   processArgs(String[])   - 
Method in class util.apps. PrintSelectColumns 
 This method will process each argument and assign any new varibles
   processArgs(String[])   - 
Method in class util.apps. ScoreChromosomes 
 This method will process each argument and assign any new variables
   processArgs(String[])   - 
Method in class util.apps. ScoreSequences 
 This method will process each argument and assign any new varibles
   processArgs(String[])   - 
Method in class util.bio.annotation. ExportIntergenicRegions 
 This method will process each argument and assign new varibles
   processArgs(String[])   - 
Method in class util.bio.annotation. ExportIntronicRegions 
 This method will process each argument and assign new varibles
   processArgs(String[])   - 
Method in class util.bio.cluster. HierarchicalClustering 
 This method will process each argument and assign new varibles
   processArgs(String[])   - 
Method in class util.bio.parsers. FetchGenomicSequences 
 This method will process each argument and assign new varibles
   processArgs(String[])   - 
Method in class util.bio.parsers.gff. ExtractGffFeatures 
 This method will process each argument and assign new varibles
   processArgs(String[])   - 
Method in class util.bio.parsers.gff. ParseWormBaseGffToUCSC 
 This method will process each argument and assign new varibles
   processArgs(String[])   - 
Method in class util.bio.parsers. SynonymMatching 
 This method will process each argument and assign any new varibles
   processArgs(String[])   - 
Method in class util.bio.seq. ConvertFasta2GCBoolean 
 This method will process each argument and assign any new varibles
   processArgs(String[])   - 
Method in class util.bio.seq. ConvertFasta2RealSeqBoolean 
 This method will process each argument and assign any new varibles
   processArgs(String[])   - 
Method in class util.bio.seq. IndexFastas 
 This method will process each argument and assign new varibles
   processArgs(String[])   - 
Method in class util.bio.seq. MakeSpliceJunctionFasta 
 This method will process each argument and assign new varibles
   processArgs(String[])   - 
Method in class util.bio.seq. MakeTranscriptome 
 This method will process each argument and assign new varibles
   processArgs(String[])   - 
Method in class util.bio.seq. OligoTiler 
 This method will process each argument and assign new varibles
   processArgs(String[])   - 
Method in class util.bio.wrappers. Primer3Wrapper 
 This method will process each argument and assign any new varibles
   processArguments(String[])   - 
Method in class util.apps. IntersectLists 
 This method will process each argument and assign new varibles
   ProcessedDataBean   - Class in  cgar.beans  Pointer to processed data file, where appropriate keep data broken down by chromosome.   ProcessedDataBean()   - 
Constructor for class cgar.beans. ProcessedDataBean 
  
   ProcessedDataTypeBean   - Class in  cgar.beans      ProcessedDataTypeBean()   - 
Constructor for class cgar.beans. ProcessedDataTypeBean 
  
   processLocalAlignment()   - 
Method in class gata.aligner. LocalAlignment 
  
   processSearchForField()   - 
Method in class bioroot. SearchBean 
  
   pseudoMedian(double[])   - 
Static method in class util.gen. Num 
 Returns a Hodges-Lehmann estimator, the median of all pairwise means.
   pseudoMedian(float[][])   - 
Static method in class util.gen. Num 
 Calculates the pseudoMedian on each replica of intensities.
   pseudoMedian(float[])   - 
Static method in class util.gen. Num 
 Returns a Hodges-Lehmann estimator, the median of all pairwise means.
   putLargestWindowArrayFirst()   - 
Method in class trans.main. MultiWindowIntervalMaker 
 Puts longest window array first and it's associated minScore.
   pValue()   - 
Method in class trans.anno. IntersectRegions 
  
   pValue(double)   - 
Method in class util.gen. Histogram 
 Returns a two tailed, p value given a threshold.
   PVALUE_ID   - 
Static variable in class edu.utah.seq.analysis. ScanSeqs 
  
   pValueLeftSide(double)   - 
Method in class util.gen. Histogram 
 Returns a one tailed, left side, p value given a threshold.
   pValueRightSide(double)   - 
Method in class util.gen. Histogram 
 Returns a one tailed, right side, p value given a threshold.
   PWMScoreWindows   - Class in  trans.roc  Scores windows for hits to a transcription factor PWM, for   RocWindow   filtering.   PWMScoreWindows(String[])   - 
Constructor for class trans.roc. PWMScoreWindows 
  
 
 


 
   
  
 
 
 
   
 
   
          Overview     
        Package   
        Class   
        Use   
          Tree     
          Deprecated     
       Index    
          Help     
   
 
 
  
 
 
 

 
  
   PREV LETTER   
   NEXT LETTER    
  
    FRAMES     
   NO FRAMES     
  
    All Classes  ');
  }
  //-->
 
 
    All Classes  
 


  
 
 
  
 

 A   B   C   D   E   F   G   H   I   J   L   M   N   O   P   Q   R   S   T   U   V   W   X   Z
